# Supplementary material for: Splice-Junction-Based Mapping of Alternative Isoforms in the Human Proteome
Source: Cell Rep. Author manuscript; Available in PMC 2020 Jan 15. (PMC6961840; doi:10.1016/j.celrep.2019.11.026)

A

sp|Q96HC4|PDLI5_HUMAN|ENSG00000163110|SE1|41190|chr4|94586444|94610304|+2|r17|T1
ANLSSSTGNVEDSFEGFR q value: 3.84e−05 Tr_novel:TRUE RefSeq_Novel:TRUE
Search result spec prec mz: 959.4392 Actual spec prec mz: 959.43921
Fragments matched per AA: 1.83 Proportion of top 20 peaks matched: 0.65

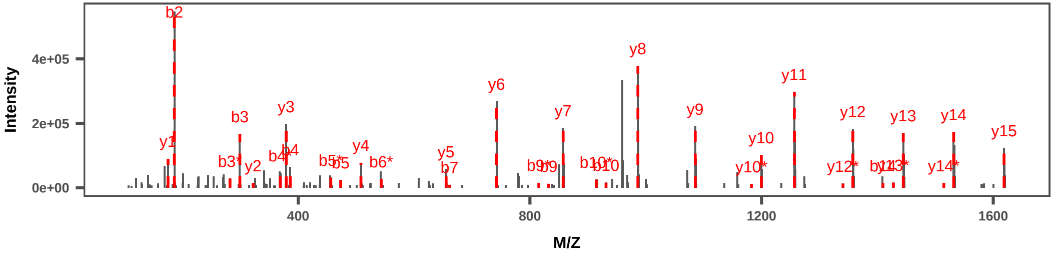

B

Scatterplot of predicted elution time
Fitting R2: 0.857
Novel peptide residual Z score: 0.546
Number of peptides: 2499

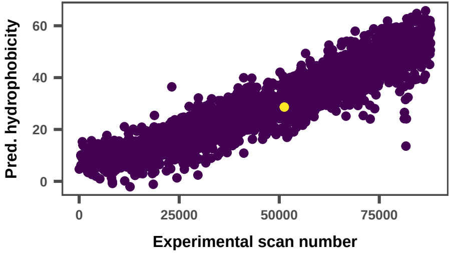

C

Distributions of residuals from best−fit line
of predicted RT vs Expt. scan number
Line: Z score of novel peptide
Z: 0.546

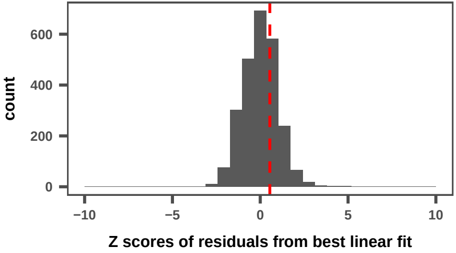

Supplement: 2 [file NIHMS1546469-supplement-2.zip › DF1/PXD006675/RightVentricle/RightVentricle_9_PDLIM5_ANLSSSTGNVEDSFEGFR.pdf]
